# Lack of Association of Initial Viral Load in SARS-CoV-2 Patients with In-Hospital Mortality

**DOI:** 10.4269/ajtmh.20-1427

**Published:** 2020-12-23

**Authors:** Anna Carrasquer, Óscar M. Peiró, Raul Sanchez-Gimenez, Nisha Lal-Trehan, Victor del-Moral-Ronda, Gil Bonet, Cristina Gutierrez, Isabel Fort-Gallifa, Carla Martin-Grau, Clara Benavent, Francesc Vidal, Alfredo Bardají

**Affiliations:** 1 Department of Cardiology, Joan XXIII University Hospital, Tarragona, Spain;; 2 Pere Virgili Health Research Institute (IISPV), Tarragona, Spain;; 3 Rovira i Virgili University, Tarragona, Spain;; 4 Clinical Laboratory, Catalan Institute of HealthTarragona, Spain;; 5 Infectious Disease Unit, Department of Internal Medicine, Joan XXIII University Hospital, Tarragona, Spain

## Abstract

Controversy exists in the literature regarding the possible prognostic implications of the nasopharyngeal SARS-CoV-2 viral load. We carried out a retrospective observational study of 169 patients, 96 (58.9%) of whom had a high viral load and the remaining had a low viral load. Compared with patients with a low viral load, patients with a high viral load did not exhibit differences regarding preexisting cardiovascular risk factors or comorbidities. There were no differences in symptoms, vital signs, or laboratory tests in either group, except for the maximum cardiac troponin I (cTnI), which was higher in the group with a higher viral load (24 [interquartile range 9.5–58.5] versus 8.5 [interquartile range 3–22.5] ng/L, *P* = 0.007). There were no differences in the need for hospital admission, admission to the intensive care unit, or the need for mechanical ventilation in clinical management. In-hospital mortality was greater in patients who had a higher viral load than in those with low viral load (24% versus 10.4%, *P* = 0.029). High viral loads were associated with in-hospital mortality in the binary logistic regression analysis (odds ratio: 2.701, 95% Charlson Index (CI): 1.084–6.725, *P* = 0.033). However, in an analysis adjusted for age, gender, CI, and cTnI, viral load was no longer a predictor of mortality. In conclusion, an elevated nasopharyngeal viral load was not a determinant of in-hospital mortality in patients with COVID-19, as much as age, comorbidity, and myocardial damage determined by elevated cTnI are.

## INTRODUCTION

The infection caused by SARS-CoV-2 has caused a global pandemic with colossal consequences.^1^ Many patients are known to remain asymptomatic or display minor symptoms after becoming infected, but others may require emergency care and hospitalization.^2^ This group of hospitalized patients is sometimes large, and in-hospital mortality is high.^3^

Mortality has been described as being related to several cardiovascular risk factors, including hypertension, diabetes, obesity, and patient comorbidity.^4,5^ It is also known that several biomarkers have prognostic implications, and especially, the presence of myocardial damage detected by the elevation of troponins is crucial in this disease.^6^ It has been described in the literature that the viral load detected in pharyngeal samples, whose detection allows the diagnosis of the infection, could be a useful prognostic marker in hospitalized patients.^7–12^ However, data available on the effects of viral load are controversial, and in most of the published articles, authors have not performed a statistical analysis adjusted for confounding variables. Our work, therefore, aims to analyze the impact of viral load on in-hospital mortality in patients with COVID-19, concerning other well-identified prognostic factors in this entity.

## METHODS

### The study, setting, design, and eligibility criteria.

No statistical methods were used to predetermine sample size. This is a retrospective observational cohort study, including reports of all patients with confirmed SARS-CoV-2 infections in a university hospital seen between March 16 and May 15, 2020. Patients were not randomized, and investigators were not blinded to outcome assessment. The vast majority of patients were first seen in the emergency service, and only those exhibiting extremely severe symptoms were admitted directly to the intensive care unit (ICU).

The patients' identification was made according to the database of determinations of the PCR test for SARS-CoV-2 in our clinical laboratory. The real-time PCR (RT-PCR) reaction was carried out in the CFX96 Touch System thermal cycler (Bio-Rad Laboratories Inc., Hercules, CA) with a commercial kit aimed at amplifying regions of the E, N, and RdRP genes (Allplex™ 2019-nCoV Assay, Seegene Inc., Seoul, South Korea). Patients were classified as positive when the E gene (screening gene) had a cycle threshold (Ct) ≤ 35 or a Ct > 35, with Ct < 40 for the confirmatory genes N and RdRP. Cycle threshold is defined as the amplification cycle’s value in which the fluorescence intensity exceeds the threshold, defined as background noise. The Ct value is inversely proportional to the number of copies of the target analyzed. Cardiac troponin I (cTnI) determinations were carried out with the immunoassay technique (high sensitivity Troponin I from Siemens, Advia Centaur^®^, Munich, Germany). The reference limit for cTnI positivity was > 47 ng/L (corresponding to the 99th percentile value with total analytical imprecision, expressed by the coefficient of variation, < 10%).

In this study, we present a collection of demographic data, cardiovascular risk factors, the reason for emergency care, clinical variables, laboratory tests, electrocardiograms, and imaging techniques (chest X-ray). In patients with several cTnI determinations, the highest value was considered. The Charlson Index (CI) score was calculated in all patients.^13^ In hospitalized patients, the need for admission to the ICU and the number of days spent being hospitalized in this unit, as well as the need for mechanical ventilation, were analyzed. The primary outcome variable was in-hospital mortality.

### Statistical analysis plan.

Categorical variables are presented in numbers and percentages, and continuous variables are presented with median and interquartile ranges. For comparisons between categorical variables, the chi-square test or Fisher’s exact test was used as appropriate, whereas the Mann–Whitney *U*-test was used when comparing continuous variables. The total sample was subdivided into two groups based on the Ct value. A Ct value exceeding 30 was considered to represent a low virus load.^11^ A binary logistic regression analysis was performed to establish the association between Ct and hospital mortality. To avoid over-fitting, this analysis was then adjusted in a multivariate model only for the following variables: age, gender, CI, and elevated cTnI. The calibration of the model was analyzed with the Hosmer–Lemeshow tests. All statistical calculations were performed using the SPSS version 22 statistical program, and a statistically significant difference was considered if *P* < 0.05.

This study is included in a broader research project on myocardial damage detected in patients seen in the emergency department and has the approval of the local Ethics Committee (Ref. CEIM: 195/2020). All patients seen in the emergency department with suspected COVID have a baseline determination of troponin and are therefore included in the general project for myocardial damage. This study is exempt from obtaining signed consent from the patients.

## RESULTS

**Figure 1.**
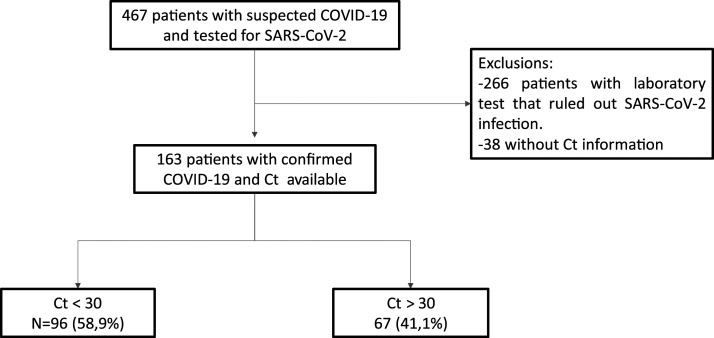
Patient flowchart.

From an initial sample of 467 patients with suspected COVID-19, 163 were included because of confirmation of the disease and available Ct (Figure 1), with a median age (interquartile range) of 67 (53–78) years. Of the total, 96 (58.9%) had Ct < 30 (high viral load) and 67 (41.1%) patients had Ct ≥ 30 (low viral load).

There were no differences regarding gender, cardiovascular risk factors, and comorbidities between patients with low viral loads and patients with high viral loads, with the exception of chronic lung disease, which was more common in patients with high viral loads (Table 1). There were no differences in symptoms, vital signs, electrocardiograms, or radiological findings between the two groups. There were also no differences in both groups’ laboratory tests except for the maximum cTnI, which showed a more significant elevation in the group with high viral load, the difference being statistically significant (Table 2). There were no differences in the need for hospital admission, admission to the ICU, or the need for mechanical ventilation in clinical management (Table 3).

**Table 1:** Demographic variables, risk factors, and comorbidity in the groups analyzed

	Total (*N* = 163)	High viral load, Ct < 30 (*N* = 96)	Low viral load, Ct => 30 (*N* = 67)	*P*-value
Demographic variables				
Age (years)	67 (53–78)	74 (59.5–81.5)	67.5 (53.7–77)	0.277
Male gender	99 (60.7)	59 (61.5)	40 (59.7)	0.821
Cardiovascular risk factors and comorbidity, *n* (%)				
Arterial hypertension	79 (48.5)	46 (51)	30 (44.8)	0.431
Diabetes mellitus	42 (25.8)	26 (27.1)	16 (23.9)	0.064
Dyslipidemia	46 (28.2)	29 (30.2)	17 (25.4)	0.5
Smoking	36 (21.1)	24 (25)	12 (17.9)	0.283
Cardiovascular history, *n* (%)				
Myocardial infarction	15 (9.2)	12 (12.5)	3 (4.5)	0.081
Heart failure	13 (8)	9 (9.4)	4 (6)	0.43
Peripheral artery disease	9 (5.5)	8 (8.3)	1 (1.5)	0.06
Cerebrovascular disease	12 (7.4)	7 (7.3)	5 (7.5)	0.967
Chronic kidney disease	17 (10.4)	12 (12.5)	5 (7.5)	0.301
Chronic lung disease	26 (16)	19 (19.8)	7 (10.4)	0.019
Dementia	15 (9.2)	8 (8.3)	7 (10.4)	0.646
Neoplasms	18 (11)	13 (13.5)	5 (7.5)	0.223
Charlson Index	1 (0–3)	2 (0–3)	1 (0–2)	0.064

Ct = cycle threshold.

**Table 2:** Main symptoms, vital signs on admission, examinations performed, and laboratory tests at the time of admission among the groups analyzed

	Total (*N* = 163)	High viral load, Ct < 30 (*N* = 96)	Low viral load, Ct = > 30 (*N* = 67)	*P*-value
Symptoms, *n* (%)				
Dyspnea	96 (58.9)	56 (58.3)	40 (59.7)	0.861
Fever	123 (76.9)	77 (80.2)	46 (71.9)	0.221
Cough	87 (54.4)	52 (54.2)	35 (54.7)	0.948
Myalgia	10 (6.3)	5 (5.3)	5 (7.8)	0.516
Diarrhea	25 (15.6)	15 (15.6)	10 (15.6)	1
Chest pain	14 (8.6)	6 (6.3)	8 (11.9)	0.202
Other symptoms	79 (48.5)	50 (52.1)	29 (43.3)	0.269
Symptom time (days)	5 (2–8)	5.5 (2–8)	5 (1.5–9)	0.496
Vital signs				
Heart rate (bpm)	86 (73–102)	82 (73–93)	84.5 (70–100)	0.566
Systolic blood pressure (mmHg)	124 (111–138)	127 (109–139)	125 (110–139)	0.565
Oxygen saturation (%)	96 (91–99)	95 (90–97)	96 (90–98)	0.229
Electrocardiogram, *n* (%)				
Atrial fibrillation	17 (11.3)	14 (15.4)	3 (5)	0.48
Left bundle branch block or right bundle branch block	6 (4)	2 (2.2)	4 (6.7)	0.169
Radiological findings, *n* (%)				
Consolidation	34 (20.9)	16 (19.8)	15 (22.4)	0.688
Frosted glass	16 (9.8)	12 (12.5)	4 (6)	0.168
Bilateral infiltrators	106 (65.4)	62 (65.3)	44 (65.7)	0.957
Laboratory tests				
Blood glucose (mg/dL)	105 (89–136)	106 (89–146)	104 (88–136)	0.87
Glomerular filtration rate (mL/minute per 1.73 m^2^)	93 (63–113)	78 (48–110)	93 (74–118)	0.191
Hemoglobin (g/dL)	12.5 (11.2–13.9)	11.8 (10.6–13.0)	12,0(11.5–13.9)	0.147
Leukocytes (×10^9^/L)	6.450 (4.710–8.910)	6.680 (4.530–9.555)	7.775 (5.557–9.042)	0.815
Lymphocytes (×10^9^/L)	0.8 (0.4–0.1)	0.6 (0.3–0.1)	0.8 (0.2–0.1)	0.542
Platelets (×10^9^/L)	212 (157–282)	190 (152–264)	241 (158–328)	0.068
D-dimer (ng/mL)	714 (431–1,679)	1,102 (530–2018)	965 (445–1947)	0.886
Lactate dehydrogenase (U/L)	278 (220–387)	308 (235–397)	269 (231–412)	0.833
C-reactive protein (mg/dL)	9 (3–16)	9 (4–17)	8.5 (3–18)	0.922
cTnI maximum (ng/L)	13 (4–35)	24 (9.5–58.5)	8.5 (3–22.5)	0.007
Elevated cTnI	29 (17.8)	20 (20.8)	9 (13.4)	0.224

Ct = cycle threshold; cTnI = cardiac troponin I.

**Table 3:** Data on hospital admission, treatments administered, and mortality among the groups analyzed

	Total (*N* = 163)	High viral load, Ct < 30 (*N* = 96)	Low viral load, Ct => 30 (*N* = 67)	*P*-value
Clinical management, *n* (%)				
Admission to hospital	141 (86.5)	85 (88.5)	56 (83.6)	0.362
Admission to ICU	31 (19)	15 (15.6)	16 (23.9)	0.186
Days in ICU	10 (0–33)	10.5 (0–35)	7 (0–25)	0.654
Mechanic ventilation	26 (16)	13 (13.5)	13 (19.4)	0.315
Acute myocardial infarction type 2	15 (9.2)	6 (6.3)	9 (13.4)	0.199
Treatment, *n* (%)				
Antibiotics*	123 (75.9)	74 (77.9)	49 (73.1)	0.485
Hydroxychloroquine	104 (64.6)	58 (61.7)	46 (68.7)	0.363
Lopinavir/ritonavir	80 (50)	47 (50)	33 (50)	1
Azithromycin	57 (35.8)	26 (28.3)	31 (46.3)	0.019
Corticosteroids	14 (8.7)	11 (11.7)	3 (4.5)	0.109
Angiotensin-converting enzyme inhibitor or angiotensin receptor blocker	20 (15.2)	11 (14.5)	9 (16.1)	0.808
Mortality, *n* (%)				
In-hospital mortality	30 (18.4)	23 (24)	7 (10.4)	0.029

Ct = cycle threshold; ICU = intensive care unit.

* Azithromycin not included.

**Figure 2.**
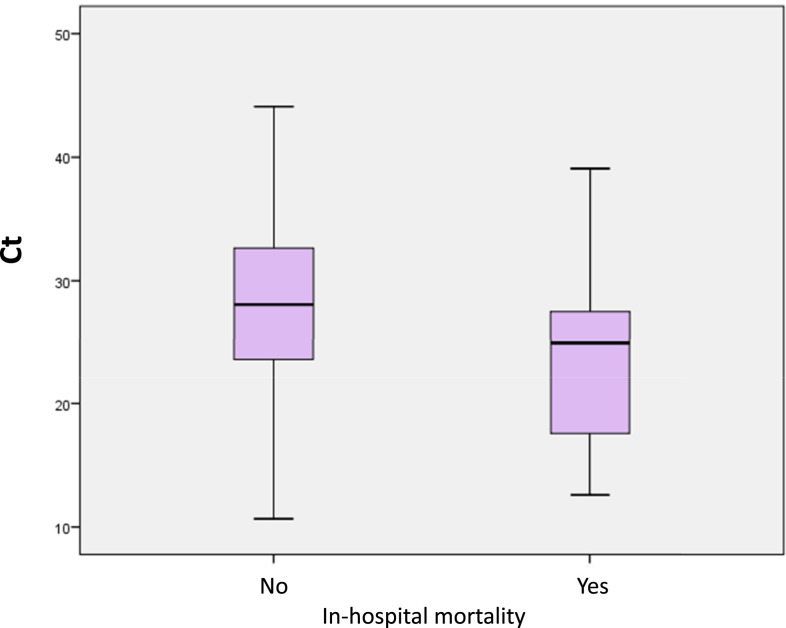
Box plot diagram of viral load cycle threshold (Ct) as a function of in-hospital mortality. This figure appears in color at www.ajtmh.org.

In-hospital mortality was higher in patients with high viral loads than in those with low viral loads (24% versus 10.4%, *P* = 0.029) (Figure 2). High viral loads were associated with in-hospital mortality in the binary logistic regression analysis (OR: 2.701, 95% CI: 1.084–6.725, *P* = 0.033). However, in an analysis adjusted for age, gender, CI, and elevated cTnI, only age, CI, and elevated cTnI remained in the model (Table 4).

**Table 4:** Predictors of in-hospital mortality by binary logistic regression

	Univariate analysis		Multivariate analysis	
	OR (95% CI)	*P*-value	OR (95% CI)	*P*-value
Age	1.067 (1.032–1.104)	< 0.001	1.044 (1.004–1.086)	0.031
Male gender	1.645 (0.701–3.864)	0.253	–	–
Charlson score	1.711 (1.355–2.160)	< 0.001	1.365 (1.058–1.760)	0.016
Cardiac troponin I elevated	8.500 (3.439–21.101)	< 0.001	4.835 (1.786–13.076)	0.002
Cycle threshold < 30	2.701 (1.084–6.725)	0.033	–	–

## DISCUSSION

This study shows that in patients admitted to a hospital with a diagnosis of SARS-CoV-2 infection by PCR of the nasopharyngeal exudate, high viral loads (considered at Ct < 30) are associated (in an unadjusted statistical model) with higher in-hospital mortality. However, when the model is adjusted for the variables that in other studies have shown a strong association with in-hospital mortality, such as age, comorbidity, and myocardial damage determined by the elevation of cTnI, the viral load did not have an independent association with in-hospital mortality.

COVID-19 has a broad clinical spectrum, and cardiovascular mortality and complications are concentrated in patients who develop a systemic condition, almost always preceded by bilateral pneumonia that progresses unfavorably. To date, numerous publications have shown that age, male gender, the existence of comorbidities, and cardiovascular risk factors are risk factors for increased mortality in SARS-CoV-2 infection.^14–16^ Likewise, other biochemical parameters play a critical role in severity stratification and prognosis.^17,18^

Nowadays, the diagnosis of SARS-CoV-2 is usually carried out by qualitative RT-PCR as is performed for the viral diagnosis of acute respiratory infections.^19^ The Ct value refers to the number of cycles in an RT-PCR assay necessary to amplify the RNA and reach a detectable level, considering that the sample is positive if its value oscillates between 0 and 40. Thus, samples with a high viral load have a low Ct value, and those with a low viral load have a higher Ct value (needing more amplification cycles).^19^ Previous publications on the 2002 SARS-CoV epidemic of SARS showed that a high viral load was related to more significant morbidity and mortality in the infective process.^20^ Therefore, our work hypothesizes whether or not the detection of the SARS-CoV-2 viral load could be used as a tool to estimate the prognosis of the disease.

Liu et al. ^10^ found that in a cohort of 76 patients, the mean viral load in severe patients was up to 60 times higher than that in mild cases. A systematic review of 18 studies carried out in the Chinese population concludes that low Ct values are significantly correlated with mortality, disease progression, and more remarkable alteration of at least one serum biomarker, including an increase in lactate dehydrogenase, a decrease of lymphocytes, and an increase in cTnI values.^21,22^ It is possible that in patients with extreme baseline severity, the viral load does affect prognosis. Hospital mortality in the Pujadas series was 32%,^23^ and in the Westblade series was 25%,^24^ thus, significantly higher than our study (18.4%). However, another study carried out in Italy, with a cohort of more than 5,000 patients, did not reveal any significant differences between viral load and disease severity.^25^ Another recent study, which included 205 patients and that performed a multivariate analysis, also found no differences in hospitalization length, the need for oxygen therapy, or mortality rates during follow-up.^9^

Viral load levels are known to be higher in the upper respiratory tract (nasopharynx and oropharynx) than in the lower respiratory tract, suggesting that the high replicability of the virus occurs in the nose and throat.^26^ In most patients with symptomatic COVID-19 infection, the viral RNA in the nasopharyngeal smear measured by Ct becomes detectable on the first day of symptoms and reaches its maximum peak within 1 week of symptom onset. PCR positivity may persist beyond three weeks after disease onset when milder cases would have a negative result, which suggests that a positive PCR result reflects only the detection of viral RNA and does not necessarily indicate the presence of a viable virus.^27^ These findings are consistent with other studies that conclude that before symptoms appear, the beginning of the infection is when the virus is reproducing the most, at least in the upper respiratory tract.^9^ This is not associated with either the duration of the symptoms or their severity. Other work published by Lavezzo et al.^28^ has shown that asymptomatic patients can spread COVID-19 in a very similar way to those that have symptoms. In our work, the probability of death in the univariate analysis was higher when the patients were older, with a higher CI score, elevated cTnI, and low Ct value results, similar to the data published by Zheng et al.^8^ In our sample, the association between mortality and Ct values was analyzed using multivariate analysis, in which viral load was no longer a predictor of mortality.

In general, SARS-CoV-2 infection has low mortality in most cases, as shown in the literature, but 10–15% of those infected suffer from the pulmonary disease, with different degrees of systemic disease leading to higher mortality,^29,30^ as has been registered in our study. When we analyzed our data, we want to note that there was a lack of knowledge about the treatments to be applied in patients with COVID-19. For example, many patients received (cardiotoxic) medications (that, in the end, do not improve outcomes), and only a few received steroids (that, in the end, improved outcomes). Our data seem to indicate that the viral load value present in respiratory samples is not the determining element in the prognosis of the COVID-19 disease, as much as age, the underlying pathologies that the patients present, and myocardial injury. Besides, the series of mechanisms triggered by the excessive activation of the immune system, which generates a cytosine storm and a pro-inflammatory and prothrombotic state, leads to higher mortality.^31^

Our study has several limitations. It was a retrospective observational study carried out in a single center with a relatively small sample size. The viral load determination was measured at the time of admission and was only obtained from respiratory samples from the upper tract. For the identification of patients, the PCR for SARS-CoV-2 was used, and although it is the method commonly used in the health field, it presents some complexity; it can have false positives and false negatives in the results. We did not have information on markers for oxygenation, like S/F (transcutaneous saturation/inspired oxygen fraction) ratio, or ROX index (ratio of oxygen saturation as measured by pulse oximetry/FIO_2_ to respiratory rate). Another limitation was not having information on the viral load in asymptomatic or mildly symptomatic patients who did not require a PCR test. Furthermore, it is possible that because of the nature of a retrospective study, we have not collected other confounding variables, which could have influenced the final results.

In conclusion, the determination of the viral load measured by the Ct value in patients with confirmed COVID-19 infection did not allow the risk of mortality to be stratified quickly and early because some other clinical factors and biomarkers do have a strong association with mortality. Higher viral load does not appear to predict a worse prognosis for the disease, but it can be used as an epidemiological marker of infectivity in mildly asymptomatic and asymptomatic outpatients.
